# Distinguishing SARS-CoV-2 infection and vaccine responses up to 18 months post-infection using nucleocapsid protein and receptor-binding domain antibodies

**DOI:** 10.1128/spectrum.01796-23

**Published:** 2023-09-22

**Authors:** Ida Jarlhelt, Laura Pérez-Alós, Rafael Bayarri-Olmos, Cecilie Bo Hansen, Maria Skaalum Petersen, Pál Weihe, Jose Juan Almagro Armenteros, Johannes Roth Madsen, Jacob Pohl Stangerup Nielsen, Linda Maria Hilsted, Kasper Karmark Iversen, Henning Bundgaard, Susanne Dam Nielsen, Peter Garred

**Affiliations:** 1 Laboratory of Molecular Medicine, Department of Clinical Immunology, Section 7631, Rigshospitalet, Copenhagen, Denmark; 2 Recombinant Protein and Antibody Unit, Copenhagen University Hospital, Copenhagen, Denmark; 3 Department of Occupational Medicine and Public Health, The Faroese Hospital System, Tórshavn, Faroe Islands, Denmark; 4 Center of Health Science, University of the Faroe Islands, Tórshavn, Faroe Islands, Denmark; 5 Department of Genetics, Stanford University School of Medicine, Stanford, California, USA; 6 Department of Emergency Medicine, Herlev-Gentofte Hospital, Copenhagen, Denmark; 7 Department of Clinical Biochemistry, Rigshospitalet, Copenhagen, Denmark; 8 Department of Clinical Medicine, Faculty of Health and Medical Sciences, University of Copenhagen, Copenhagen, Denmark; 9 The Heart Center, Department of Cardiology, Rigshospitalet, Copenhagen, Denmark; 10 Viro-immunology Research Unit, Department of Infectious Diseases, Section 8632, Rigshospitalet, Copenhagen, Denmark; Karolinska Institutet, Stockholm, Sweden

**Keywords:** SARS-CoV-2, nucleocapsid protein, spike protein, RBD, sandwich antibody ELISA, antibody duration

## Abstract

The prediction of the durability of immunity against COVID-19 is relevant, and longitudinal studies are essential for unraveling the details regarding protective SARS‐CoV‐2 antibody responses. It has become challenging to discriminate between COVID-19 vaccine- and infection-induced immune responses since all approved vaccines in Europe and the USA are based on the viral spike (S) protein, which is also the most commonly used antigen in immunoassays measuring immunoglobulins (Igs) against SARS-CoV-2. We have developed a nucleocapsid (N) protein-based sandwich ELISA for detecting pan anti-SARS-CoV-2 Ig with a sensitivity and specificity of 97%. Generalized mixed models were used to determine the degree of long‐term humoral immunity against the N protein and the receptor-binding domain (RBD) of the S protein in a cohort of infected individuals to distinguish between COVID-19 vaccine- and infection-induced immunity. N-specific waning could be observed in individuals who did not experience reinfection, while individuals who experienced reinfection had a new significant increase in N-specific Ig levels. In individuals that seroconverted without a reinfection, 70.1% remained anti-N seropositive after 550 days. The anti-RBD Ig dynamics were unaffected by reinfection but exhibited a clear increase in RBD-specific Ig when vaccination was initiated. In conclusion, a clear difference in the dynamics of the antibody response against N protein and RBD was observed over time. Anti-N protein-specific Igs can be detected up to 18 months after SARS-CoV-2 infection allowing long-term discrimination of infectious and vaccine antibody responses.

**IMPORTANCE:**

Longitudinal studies are essential to unravel details regarding the protective antibody responses after COVID-19 infection and vaccination. It has become challenging to distinguish long-term immune responses to SARS-CoV-2 infection and vaccination since most approved vaccines are based on the viral spike (S) protein, which is also mostly used in immunoassays measuring immunoglobulins (Igs) against SARS-CoV-2. We have developed a novel nucleocapsid (N) protein-based sandwich ELISA for detecting pan-anti-SARS-CoV-2 Ig, exhibiting high sensitivity and specificity. Generalized mixed models were used to determine long‐term humoral immunity in a cohort of infected individuals from the Faroe Islands, distinguishing between COVID-19 vaccine- and infection-induced immunity. A clear difference in the dynamics of the antibody response against N protein and S protein was observed over time, and the anti-N protein-specific Igs could be detected up to 18 months after SARS-CoV-2 infection. This enables long-term discrimination between natural infection and vaccine-dependent antibody responses.

## INTRODUCTION

The first case of coronavirus disease 2019 (COVID-19) was reported in December 2019, and the cumulative number of confirmed COVID-19 cases has now exceeded 750 million worldwide (1). COVID-19 is caused by severe acute respiratory syndrome coronavirus 2 (SARS-CoV-2), and vaccination has now been implemented globally, which appeared early on to be highly effective in reducing disease severity and in eliciting highly protective immune responses (2, 3). However, evidence suggests that vaccination is not as effective in preventing viral transmission, at least not for the omicron variant (4), and breakthrough infections among vaccinated individuals, as well as reinfections among those previously infected, are continual (5, 6).

The SARS-CoV-2 viral genome encodes four structural proteins, namely, spike (S), envelope (E), membrane (M), and nucleocapsid (N) (7). Much attention has been focused on the S protein because it contains the receptor-binding domain (RBD) that interacts with ACE-2 host receptor (8). The S protein has, throughout the pandemic, been frequently used as an antigen in many immune assays to measure infection-induced antibody immunity (9). However, since all approved vaccines in Europe and the USA are based on the S protein, it is no longer possible to discriminate between natural infection and vaccine-induced immunity using S protein or RBD as an antigen. The N protein is immunogenic and is highly expressed in virus-infected cells (10). In addition, the N protein is not used as an antigen in the currently approved vaccines, making it a good candidate and target antigen to detect infection in vaccinated individuals (11, 12). However, most N protein assays are based on the direct detection of antibody isotypes bound to N protein, and these assays are typically hampered by low sensitivity (13, 14). A commercial assay, the Elecsys Anti-SARS-CoV-2 N protein assay (Roche Diagnostics), has solved this problem using the sandwich principle where N protein is used for both capture and detection, allowing the contribution of all the N protein-specific immunoglobulin (Ig) isotypes and thereby increasing the assay sensitivity (15). It should be mentioned that another commercial platform measures total N-specific Ig, namely, the Platelia SARS-CoV-2 Total Ab assay (Bio-Rad), and this assay is nevertheless not available in Europe. The downside is that the commercial assays require expensive analytical equipment present only in specialized laboratories. Thus, the development of a flexible, simple, and sensitive anti-N protein total Ig assay with broad availability would be valuable. We therefore sought to develop an open anti-N protein pan Ig ELISA-based platform as a proxy for previous SARS-CoV-2 infections. The sandwich principle utilizes that antibodies have multiple binding sites that allow for capturing between two antigens. The same principle has been widely employed for RBD both in commercial and in-house constructed assays (16).

Nevertheless, whether anti-N protein Ig in general constitutes a robust measurement of natural infection over time has been debated. Studies suggest that the developed antibodies against the N protein wane more rapidly than antibodies against the S protein and become undetectable within 6–9 months following infection (17, 18). This possibly leads to a marked underestimation of the true proportion of people with previous infection. Because of this uncertainty, we (i) developed an open ELISA sandwich platform that can be used widely on low-cost equipment to monitor SARS-CoV-2 infectious responses and (ii) addressed how long an anti-N-specific immune response can be detected to discriminate between the long-term effect of hybrid immunity in relation to infectious and vaccine responses.

## MATERIALS AND METHODS

### Study participants

To validate the assay, we included 375 SARS-CoV-2 positive individuals from a prospective observational study described previously (19). Briefly, previously infected healthcare professionals from Rigshospitalet and Herlev-Gentofte University Hospital (Capital Region of Denmark) volunteered to participate in a prospective longitudinal observational study to determine the dynamics of the Ig levels after SARS-CoV-2 infection and/or vaccination. All individuals were confirmed positive by reverse transcription polymerase chain reaction (RT-PCR) testing on material from oropharyngeal swabs. Samples were collected between 25 and 500 days after a positive PCR test. This protocol was approved by the Regional Scientific Ethics Committee of the Capital Region of Denmark (H-20079890). Also, 375 samples from anonymous blood donors (collected in 2013 and 2014) were included as pre-pandemic healthy controls. The study was approved by the Regional Health Ethics Committee of the Capital Region of Denmark (H2-2011-133). Collectively, these samples are referred to as cohort I, the validation cohort. To ensure good performance of the developed assay, the 375 positive samples from cohort I were analyzed and compared with a double-antigen (N protein) sandwich commercial assay (Elecsys Anti-SARS-CoV-2 assay). In addition, to validate the assay in another cohort and to compare our newly developed N protein sandwich ELISA to the performance of the already established in-house RBD sandwich ELISA, we analyzed 200 SARS-CoV-2 anti-RBD-positive samples and 200 SARS-CoV-2 anti-RBD-negative samples obtained from the medical students of the University of Copenhagen. The 400 students are a subset of a cohort already described elsewhere (20). Briefly, sera were collected during the fall of 2020 before the implementation of the vaccine campaign. A double-antigen RBD sandwich ELISA (S-ELISA)-based commercial assay (Beijing Wantai Biological Pharmacy Enterprise) was used to determine seropositivity by measuring total Ig against SARS-CoV-2 RBD. The protocol complied with the Declaration of Helsinki II and was approved by the Regional Ethics Committee of the Capital Region (H-20055767) and by the Danish Data Protection Authorities (P-2020–92). The cohort is referred to as cohort II, the performance cohort.

We used a longitudinal study cohort collected at the Faroe Islands for the long-term evaluation of N-specific immune responses. The Faroe Islands is a self-governing part of the Danish Kingdom. Within the Faroe Islands, all individuals who were infected with SARS-CoV-2 during the first wave (3 March to 22 April 2020) and the second wave (3 August to 25 December 2020) were invited to participate in a prospective longitudinal observational study that has been described elsewhere (21, 22). All individuals were confirmed positive by RT-PCR testing on material from oropharyngeal swabs. The vaccine rollout in the Faroe Islands began on 30 December 2020, with the Pfizer-BioNTech vaccine (BNT162b2) as the only vaccine option. A total of 100 individuals from the second wave, which all had five consecutive serum samples taken during the period from infection onset (summer 2020) until spring 2022, were included in the study cohort. All participants provided informed written consent, and the Faroese Research Ethical Committee and the Faroese Data Protection Agency approved the study. The cohort is referred to as cohort III, the study cohort. An overview of the cohorts can be found in Table S1.

### Buffers

The following buffers were used: PBS (10.1 mM Na_2_HPO_4_, 1.5 mM KH_2_PO_4_, 137 mM NaCl, 2.7 mM KCl), PBS-T [PBS, 0.05% Tween 20 (8221840050; Merck)], and dilution buffer [PBS-T, 5 mM EDTA (EDS-500G; Merck), 5% skim milk (70166; Merck)].

### SARS-CoV-2 antigens

The full-length sequence of N protein from the ancestral SARS-CoV-2 strain (QLD29192.1) was synthesized by GeneArt (Thermo Fisher Scientific) into a pcDNA3.4 plasmid downstream of the human serum albumin signal peptide and upstream of a tandem 8xHis-Avi tag using glycine-serine linkers (GS-HHHHHHHH-GSG-GLNDIFEAQKIEWHE). The production, purification, and biotinylation have been described elsewhere, as well as the production of recombinant S protein RBD (23).

### The development of the anti-N protein Ig sandwich-ELISA

The anti-N protein Ig S-ELISA measuring total Ig levels against N protein was optimized with regard to dilution range, blocking buffers, and variations in incubation times. In the final assay, Nunc MaxiSorp flat-bottom 96-well plates (442404; Thermo Fisher Scientific) were coated with 0.5 µg/mL N protein in PBS and incubated overnight at 4°C. Clinical samples and control sera were diluted in the ratio 1:2 in the dilution buffer and incubated for 1 h shaking at room temperature (RT). A positive control pool consisted of sera drawn from N-positive individuals, and a negative control pool consisted of sera drawn from healthy individuals before the SARS-CoV-2 emergence. Ig bound to SARS-CoV-2 N protein was detected using 40 ng/mL biotinylated N protein diluted in PBS-T + 5% cow serum and incubated for 1 h shaking at RT. Pierce HRP-conjugated high-sensitivity streptavidin (21130; Thermo Fisher Scientific) was used for detection, diluted 1:16,000 in PBS-T and incubated for 1 h shaking at RT. TMB One substrate (4380A; Kementec) was applied and allowed to react for 20 min. The reaction was stopped with 0.3 M H_2_SO_4_, and the optical density (OD) was measured at 450 nm using a reference wavelength of 630 nm on a Synergy HT absorbance reader (BioTek Instruments). The total volume of each reagent in all incubation steps was 100 µL/well. Plates were washed three times with 400 µL/well PBS-T between incubation steps. All shaking steps were performed using an orbital shaker at 800 rpm.

### Assay validation

The final conditions for the developed anti-N protein Ig S-ELISA were chosen based on the OD of the absorbance signal and the signal-to-noise (S/N) ratio between a sample and a negative control sample. Specificity and sensitivity were calculated based on a receiver operating characteristic (ROC) curve, and the cutoff was selected. Intra-assay variation was evaluated by calculating the coefficient of variation (CV), representing the percentage of individual CVs for all the duplicates in 45 samples. Inter-assay variation was evaluated by calculating the CV of samples in 5× replicates on two plates at five different occasions. To assess the dilution linearity and thereby the range of the assay, three serum samples containing high, intermediate, and low levels of antibodies against N protein were analyzed, together with a pre-pandemic negative control. In addition, the dilution linearity of an in-house-produced monoclonal mouse antibody against full-length N protein was likewise evaluated both independently and spiked into a pre-pandemic negative control. The parallelism between serum and plasma samples was evaluated by comparing 60 pairs of serum and EDTA plasma samples. The same 60 samples were additionally analyzed and compared in a manual setup versus automatization in a high-throughput setup handled by the Biomek FX Automated Workstation (Beckman Coulter, Brea, CA).

### Assay performance

#### N-specific S-ELISA vs RBD-specific S-ELISA

To investigate the accordance between the newly developed N protein assay and our RBD sandwich ELISA (as described below), 200 RBD-positive samples and 200 RBD-negative samples were analyzed in the N protein sandwich ELISA. Also here, a ROC curve was used to investigate the sensitivity and specificity of the assay in another cohort.

#### S-ELISA vs direct ELISA

To compare the performance of the newly established sandwich ELISA with a direct setup, a subset of 215 samples from cohort I were subjected to direct ELISA measuring N-specific IgG, IgM, and IgA. Briefly, Nunc MaxiSorp flat-bottom 96-well plates (442404; Thermo Fisher Scientific) were coated with 0.5 µg/mL N protein in PBS and incubated overnight at 4°C. Clinical samples and control sera were diluted in the ratio 1:100 in the dilution buffer and incubated for 1 h shaking at RT. Abs bound to SARS-CoV-2 Ags were detected using 0.5 µg/mL HRP-conjugated polyclonal rabbit Abs against human IgG (P0214), IgM (P0215), or IgA (P0216) (all from Agilent Technologies, Santa Clara, CA) diluted in PBS-T and incubated for 1 h shaking at RT. TMB One substrate (4380A; Kementec) was applied and allowed to react for 5 min for IgG and 10 min for IgA and IgM. The reaction was stopped with 0.3 M H_2_SO_4_, and the optical density (OD) was measured at 450 nm using a reference wavelength of 630 nm on a Synergy HT absorbance reader (BioTek Instruments). A ROC curve was used to calculate the best-fit cutoff to estimate the performance of the assay(s).

#### In-house N-specific S-ELISA vs commercial N-specific assay

To compare the performance of the newly established anti-N protein Ig assay with a commercial anti-N protein assay, 375 positive samples from cohort I were analyzed in the Elecsys anti-N protein Ig assay from Roche Diagnostics against our N protein S-ELISA. The Elecsys assay was performed using the COBAS 8000 platform (e801 module) following the manufacturer’s instructions.

### RBD sandwich ELISA

Levels of total Ig against RBD were measured using a validated ELISA-based assay as described elsewhere with minor modifications (23). Briefly, Nunc MaxiSorp flat-bottom 96-well plates (442404; Thermo Fisher Scientific) were coated with 0.5 µg/mL RBD in PBS overnight at 4°C. Plates were blocked with PBS-T for 1 h at RT. Samples were applied to plates diluted in the ratio of 1:2 in PBS-T and incubated for 1 h shaking at RT. Biotinylated RBD was then added in a concentration of 0.5 µg/mL in PBS-T and incubated for 1 h shaking at RT. Anti-SARS-CoV-2 RBD Igs were detected using Pierce HRP-conjugated high-sensitivity streptavidin, diluted in the ratio of 1:16,000 in PBS-T and incubated for 1 h shaking at RT. TMB One was used as a substrate and allowed to react for 5 min. The reaction was stopped with 0.3 M H2SO4, and the OD was measured at 450 nm and at 630 nm using a Synergy HT absorbance reader (BioTek Instruments). Plates were washed three times with PBS-T between incubation steps. The development and assay validation of the RBD S-ELISA has been described previously (23). Nonetheless, the assay was later revalidated after the introduction of the minor assay modifications, and the final performance of the modified assay is found in Fig. S1.

### Statistical analyses and modeling

Statistical analyses and modeling were performed using R (version 4.1.0 for Windows, R Foundation for Statistical, Computing). ROC curve analysis was performed using GraphPad Prism version 9.2.0 (GraphPad Software, La Jolla, CA). The anti-N protein Ig levels measured by the novel S-ELISA were correlated with the levels measured in the Elecsys anti-N protein Ig assay using the Spearman’s rank correlation test. The parallelism between assay conditions, including blood preparation and assay handling, was analyzed using the Spearman rank correlation test. The changes in the levels of total Ig against N protein dynamics were modeled from the days of infection onset and up to 582 days using a generalized linear mixed model (GLMM) with Gaussian distribution. The days from infection onset were represented with two natural cubic splines to allow the modeling of non-linear trends. Changes in levels of total Ig against RBD dynamics were modeled from the days of infection onset and up to 582 days using a GLMM. The days from infection onset were represented with three natural cubic splines to allow the modeling of non-linear trends. Interactions were analyzed between days and reinfection status and days and vaccination. Age groups and sex were included in the analyses as covariates. During analysis, anti-N protein Ig levels were log10 transformed and back transformed when reported. *P*-values reported from the fitted GLMMs were calculated using Type II Wald chi-square tests. *P*-value <0.05 was considered significant.

## RESULTS

### Development of a sandwich ELISA for the detection of N protein-specific Ig

**Fig 1:**
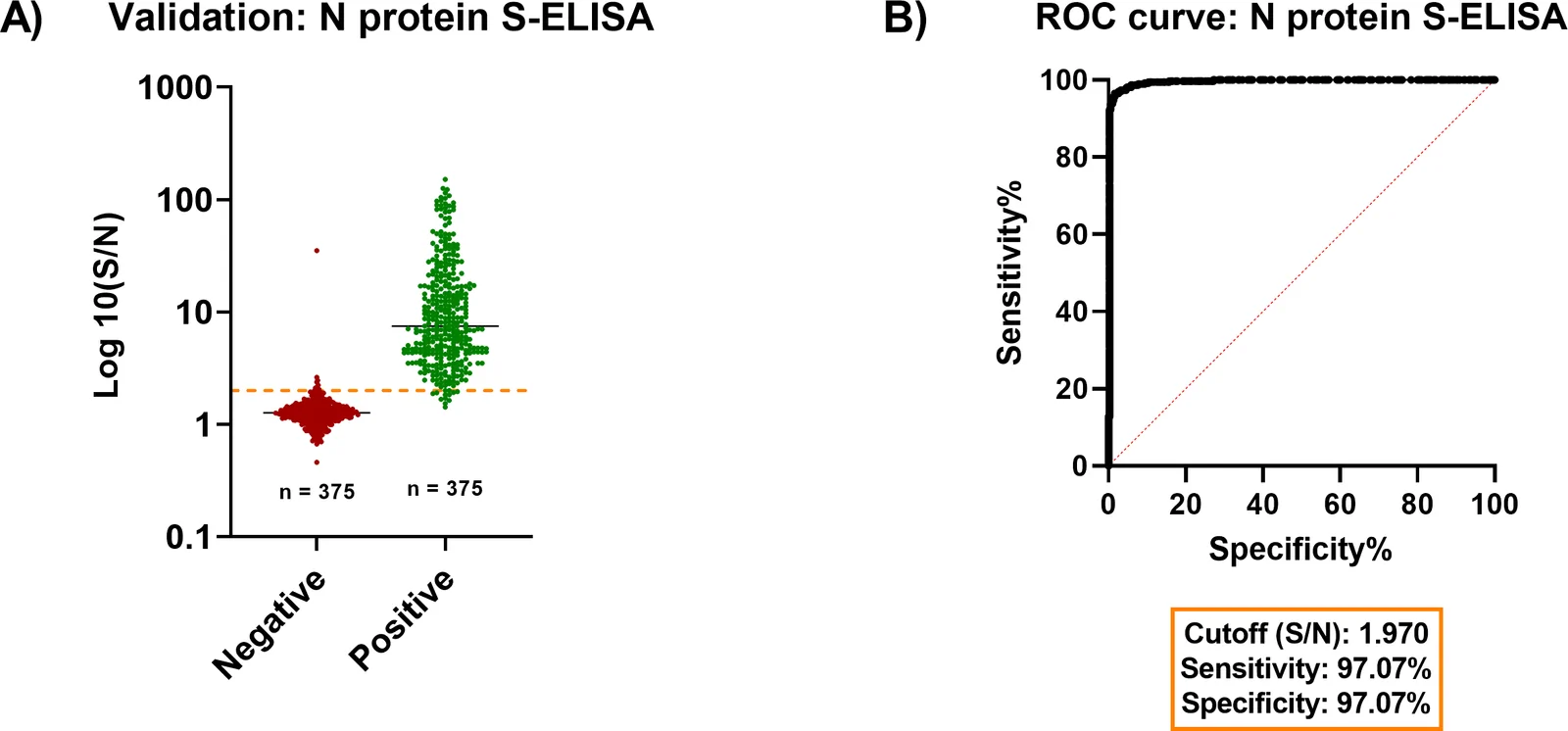
Assay validation of the newly developed anti-N protein Ig sandwich ELISA. The newly developed S-ELISA was validated using 375 PCR-confirmed SARS-CoV-2-infected individuals and 375 pre-pandemic negative controls, which were subjected to antibody detection against N protein (**A**). ROC curve analysis was used to evaluate the performance of the N-specific sandwich ELISA setup detecting total Ig (**B**). Horizontal dashed line represents the positivity threshold. S/N, signal to noise.

A total of 750 samples, 375 SARS-CoV-2 PCR positive and 375 pre-pandemic negative, were subjected to total Ig measurements in the N protein S-ELISA (Fig. 1A). A ROC curve was used to calculate the best-fit cutoff to estimate the performance of the assay. The anti-N protein Ig S-ELISA was performed with a 97.07% sensitivity and 97.07% specificity with a cutoff of 1.970 (S/N) (Fig. 1B). The intra- and inter-assay variations were found to be acceptable (CV < 10%) (Table S2). The dilution linearity of three serum samples with high, intermediate, and low levels of N-specific antibodies, alongside a negative control, is shown in Fig. S2. We additionally analyzed the linearity of an in-house-produced antibody against N protein both independently and spiked into the pre-pandemic negative control. The dilution linearity was found to be acceptable, and the latter experiment confirms that the signal from a pre-pandemic negative control can be recovered by spiking-in N-specific antibodies. The S-ELISA assay was suitable for both manual handling and automatization in a high-throughput setup in 96-well format, with the two approaches correlating significantly (*r* = 0.9763, *P* < 0.0001; Fig. S3A). Moreover, a significant correlation (*r* = 0.89, *P* < 0.0001; Fig. S3B) was observed between EDTA plasma and serum.

### Assay performance

**Fig 2:**
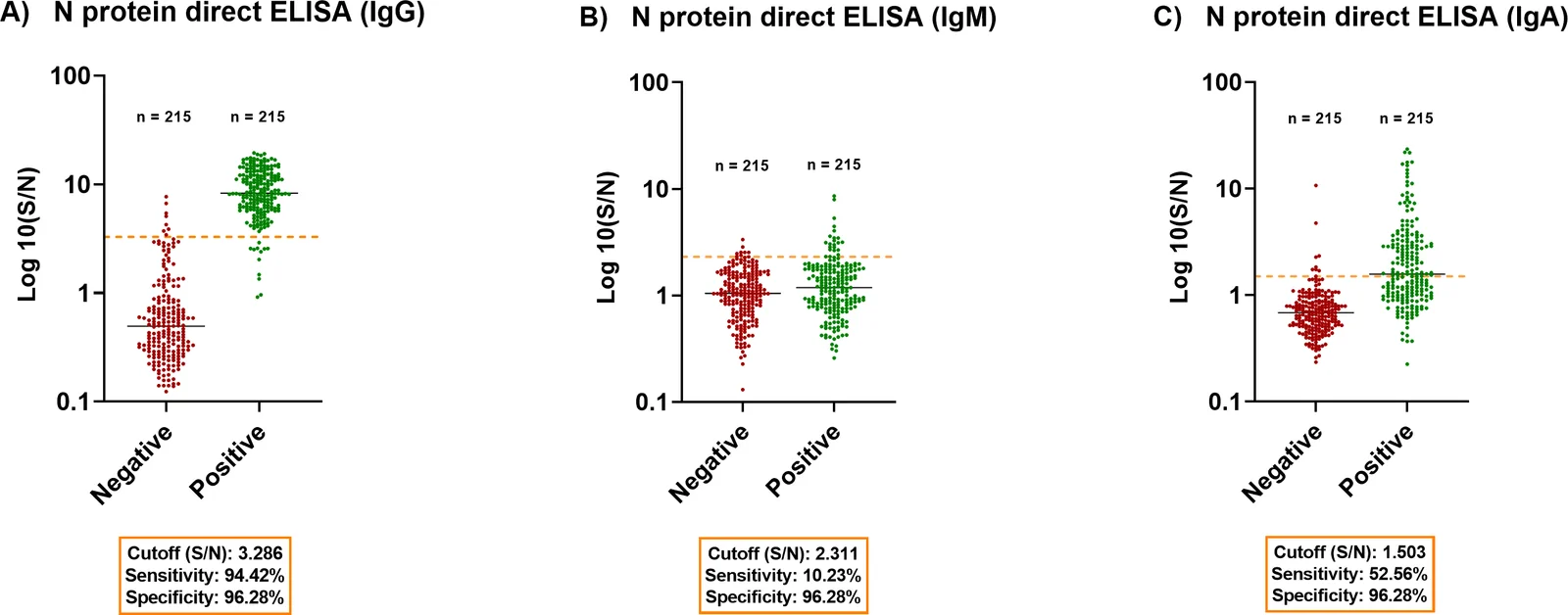
Performance in a N protein-specific direct ELISA setup; 215 PCR-confirmed SARS-CoV-2-infected individuals and 215 pre-pandemic negative controls were used for the validation of a N-specific direct ELISA setup. The samples were analyzed in a direct assay detecting IgG (**A**), IgM (**B**), or IgA (**C**) against N protein. A ROC curve analysis was used to evaluate the cutoff, sensitivity, and specificity of each assay. Horizontal dashed line represents the positivity threshold. S/N, signal to noise.

**Fig 3:**
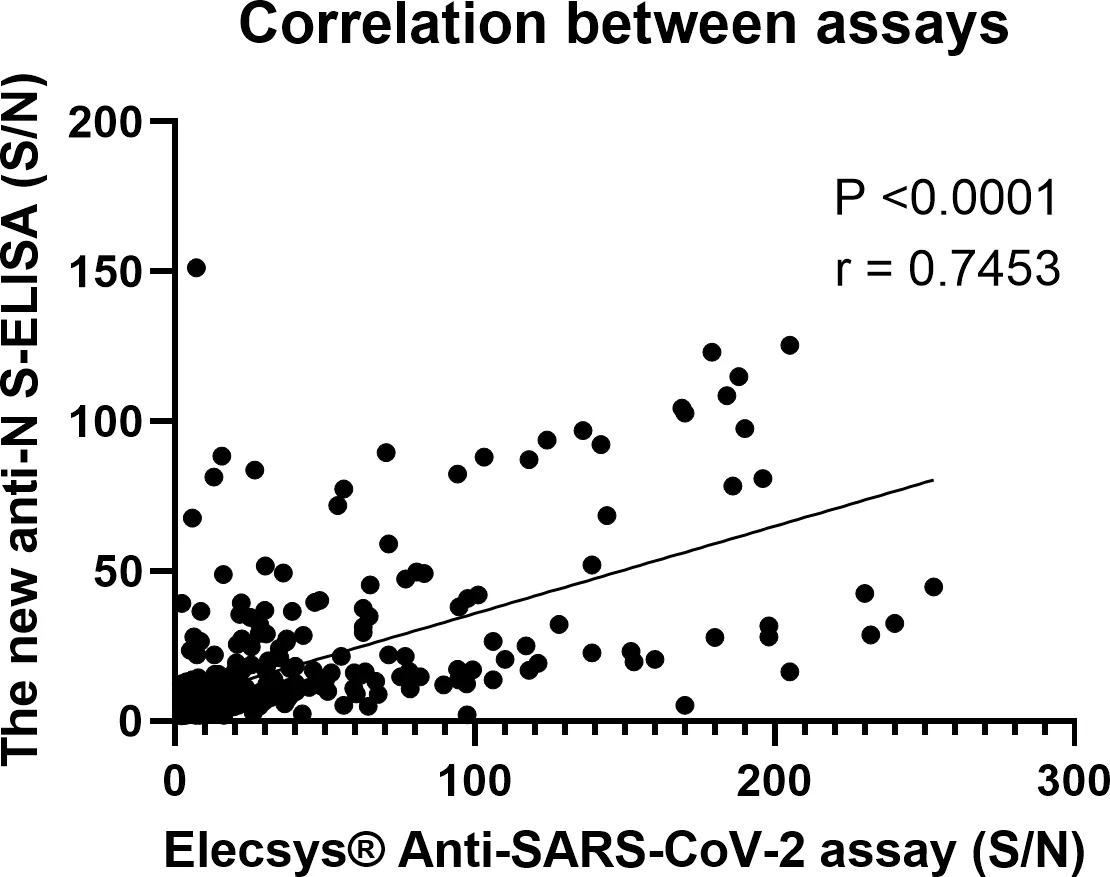
Performance of the anti-N protein Ig sandwich ELISA compared to the Elecsys assay; 375 PCR-positive samples were analyzed in the commercial Elecsys Anti-SARS-CoV-2 assay and compared to the data obtained by our newly developed N S-ELISA. The parallelism between the performance of the two assays was evaluated using a Spearman’s rank correlation test. *P* < 0.05 was considered significant. S/N, signal to noise.

In total, 200 RBD-positive samples and 200 RBD-negative samples, referred to as the performance cohort, were analyzed in the N protein sandwich ELISA to investigate the performance of the newly developed assay against our in-house RBD sandwich ELISA (Fig. S4). The N- specific S-ELISA was performed with a sensitivity of 97% and a specificity of 98.5% when applying the performance cohort, indicating an almost complete accordance in seropositivity between RBD and N protein-specific antibodies. To investigate how the N-specific S-ELISA was performed against a direct ELISA setup, a subset of the validation cohort was additionally analyzed in a direct assay measuring N-specific IgG, IgA, and IgM (Fig. 2). With 215 PCR-positive samples and 215 pre-pandemic samples, the anti-N protein IgG direct ELISA was performed with a sensitivity of 94.4% and specificity of 96.28% (Fig. 2A). When choosing the same specificity for the IgM and the IgA assay, the sensitivity was 10.2% and 52.6%, respectively (Fig. 2B and C). In addition, to evaluate the performance of the anti-N protein Ig S-ELISA against a commercial and validated N protein SARS-CoV-2 serological assay (Elecsys Anti-SARS-CoV-2 assay), we analyzed the 375 positive samples from cohort I. The resulting values were significantly correlated (Spearman’s rank, *r*  =  0.745, *P* < 0.0001), illustrating that the newly developed assay detects comparable Ig levels as the Elecsys (Fig. 3).

### Waning of N and RBD-specific Ig in infected individuals

Levels of SARS-CoV-2 Ig against protein N and RBD were measured in plasma samples from 100 individuals previously infected with SARS-CoV-2, at five consecutive time points from summer 2020 until spring 2022 (21). The characteristics of the study participants are described in Table 1. Disease onset was defined as the day of symptom onset, or if asymptomatic, the day of positive RT‐PCR testing. The cohort had an even sex distribution (52% and 48%, respectively) with a median age of 45 (IQR: 26–54) years. Most of the individuals reported at least one symptom during infection (80%) and were vaccinated with at least one dose of the BNT162b2 vaccine during the period of the study (86%) (Table 1). The median days from disease onset to the first blood sampling were 27 days (IQR: 25–30), and the median days from disease onset to the first vaccination dose were 287 days (IQR: 255–328). Some individuals were reinfected with SARS-CoV-2 (31%) at the beginning of 2022, all confirmed by a new RT‐PCR test. In this group of reinfected individuals, the median days from the latest infection to the second positive RT-PCR test were 529 days (IQR: 509–537).

**TABLE 1:** Demographic characteristics of the longitudinal cohort

	Not reinfected (*n* = 69)	Reinfected (*n* = 31)	Total (*n* = 100)
Sex			
Male	34 (49.3%)	14 (45.2%)	48 (48.0%)
Female	35 (50.7%)	17 (54.8%)	52 (52.0%)
Age (years)			
Median (IQR^*c*^)	49.7 (31.0–56.8)	30.3 (22.3–44.8)	44.8 (26.1–53.8)
Age groups (years)			
<30	16 (23.2%)	15 (48.4%)	31 (31.0%)
>30–50	20 (29.0%)	9 (29.0%)	29 (29.0%)
>50	33 (47.8%)	7 (22.6%)	40 (40.0%)
Symptoms in the acute phase^*a*^			
Yes	53 (76.8%)	27 (87.1%)	80 (80.0%)
No	5 (7.2%)	0 (0%)	5 (5.0%)
Not available	11 (15.9%)	4 (12.9%)	15 (15.0%)
Vaccination			
Vaccinated^*b*^	67 (97.1%)	19 (61.3%)	86 (86.0%)
Unvaccinated	2 (2.9%)	12 (38.7%)	14 (14.0%)
Vaccine doses			
0	2 (2.9%)	12 (38.7%)	14 (14.0%)
1	4 (5.8%)	0 (0%)	4 (4.0%)
2	27 (39.1%)	11 (35.5%)	38 (38.0%)
3	36 (52.2%)	8 (25.8%)	44 (44.0%)
Days from disease onset to first vaccine dose			
Median (IQR)	284 (249–328)	300 (266–320)	287 (255–328)
Days from disease onset to blood sampling Median (IQR)			
First sampling	27 (25–30)	26 (24–29)	27 (25–30)
Second sampling	125 (110–132)	129 (118–131)	126 (112–132)
Third sampling	203 (177–221)	217 (192–223)	212 (173–222)
Fourth sampling	304 (267–313)	308 (287–314)	306 (269–314)
Fifth sampling	548 (530–573)	561 (545–573)	554 (532–573)

^*a*^ At least one symptom reported.

^*b*^ Vaccinated with at least one dose of the BNT162b2 vaccine.

^*c*^ IQR, interquartile range.

**Fig 4:**
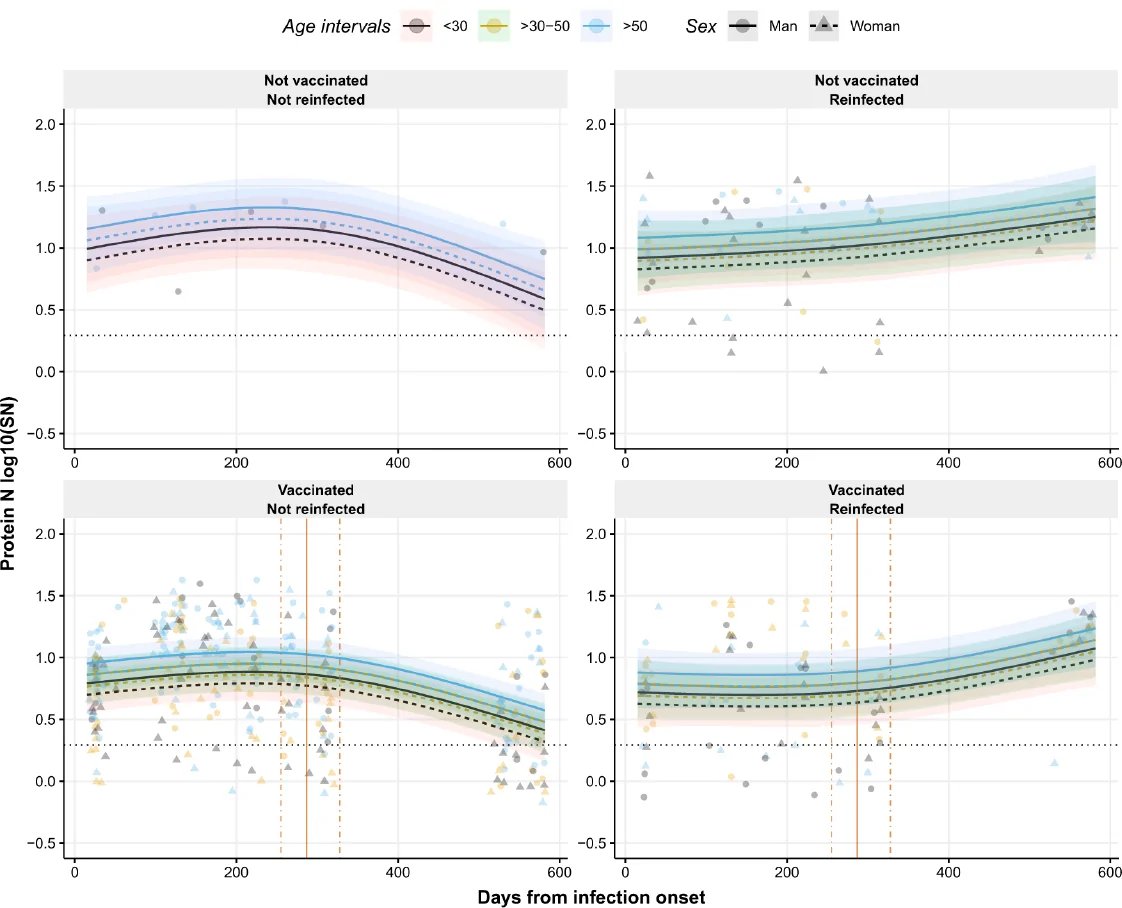
The waning of the antibody response against SARS-CoV-2 N protein. Distribution of total Ig levels against N protein over time (days from the infection onset) in individuals not vaccinated not reinfected (top left), not vaccinated but infected (top right), not vaccinated but reinfected (bottom left), and both vaccinated and reinfected (bottom right). Data are represented in log10[signal-to-noise (S/N) values]. Circles and triangles represent the observed levels of circulating N total antibodies in males and females, respectively. Solid and dashed lines represent the predicted levels of circulating N total antibodies calculated by the model in males and females, respectively. Black, yellow, and blue colors represent individuals with age <30, 30–50, and >50 years, respectively. Horizontal black dotted line represents the threshold for assay positivity. Vertical solid line represents the median days from disease onset where first vaccination dose was given, and vertical dash-dotted lines represent the IQR. Center for the confidence interval is the predicted (mean) values, and shadowed areas represent the 95% confidence interval.

**Fig 5:**
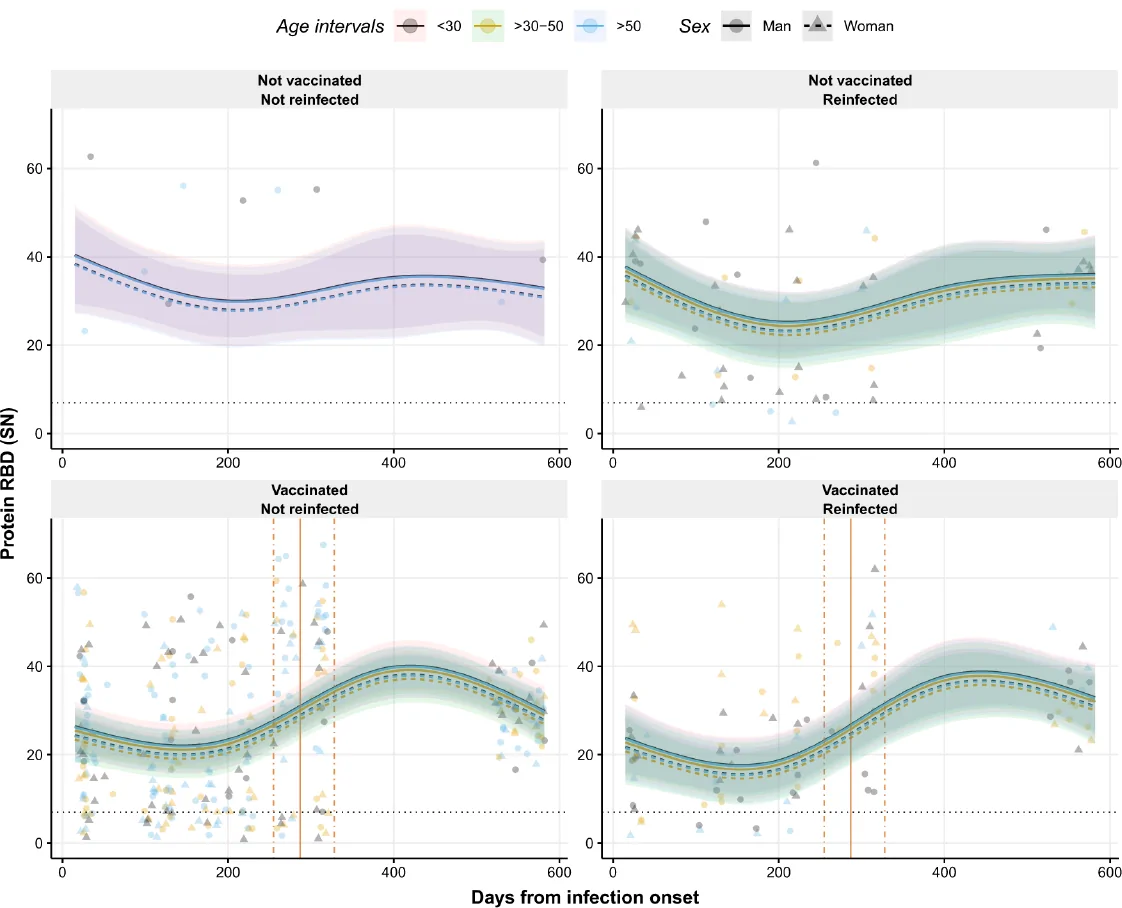
The waning of the antibody response against SARS-CoV-2 RBD. Distribution of total Ig levels against RBD over time (days from the infection onset) in individuals not vaccinated not reinfected (top left), not vaccinated but infected (tog right), not vaccinated but reinfected (bottom left), and both vaccinated and reinfected (bottom right). Data are represented in signal-to-noise (S/N) values. Circles and triangles represent the observed levels of circulating RBD total antibodies in males and females, respectively. Solid and dashed lines represent the predicted levels of circulating RBD total antibodies calculated by the model in males and females, respectively. Black, yellow, and blue colors represent individuals with age <30, 30–50, and >50 years, respectively. Horizontal black dotted line represents the threshold for assay positivity. Vertical solid line represents the median days from disease onset where first vaccination dose was given, and vertical dash-dotted lines represent the IQR. Center for the confidence interval is the predicted (mean) values, and shadowed areas represent the 95% confidence interval.

By using a GLMM with natural cubic splines, we developed two non-linear models to evaluate the dynamics of circulating Ig against N protein or RBD over time after disease onset. The models were adjusted for sex and age groups (<30, 30–50, >50 years), and RT-PCR confirmed the reinfection and vaccination status. There were no individuals within the age group of 30–50 years, who were unvaccinated and without re-infection, and therefore, no observed data to create a prediction of these conditions. Over time, total anti-N Ig level dynamics significantly differed within individuals with and without confirmed reinfection (*P* < 0.0001; Fig. 4). Waning could be observed in individuals without a SARS-CoV-2 reinfection regardless of vaccination status, while individuals who experienced reinfection at the beginning of 2022 had a new increase in the levels of N-specific Ig. Vaccination status did not significantly influence N protein-specific Ig level dynamics over time (*P* = 0.649). Age groups and sex were not significantly associated with N-specific Ig level dynamics. The antibodies against RBD appeared to decline during the first month after infection, as previously reported (21). An increase in RBD-specific Ig levels was observed when vaccination was initiated at the end of 2020, followed by a peak and new waning period until the final sample was taken during spring 2022 (Fig. 5, lower panel). Vaccination had a significant impact on anti-RBD Ig levels over time (*P* = 0.015; Fig. 4). On the contrary, reinfection status did not influence the total Ig against RBD levels dynamics over time (*P* = 0.261; Fig. 4). Age groups and sex were not significantly associated with RBD-specific Ig-level dynamics.

### N-specific seropositivity over time

Regarding the overall seroconversion after natural infection, 95% of the individuals seroconverted to anti-N Ig, while 97% seroconverted to anti-RBD Ig before vaccination. We could investigate seropositivity over time within the group of individuals that did not experience reinfection (Table 2). Two individuals without a positive N-specific response at any time point, most likely due to false-positive PCR testing, were excluded from this analysis. The proportion of anti-N protein seropositive individuals appears to increase during the initial 100 days after disease onset, reaching a plateau with 98.5% individuals positive for Igs against N protein approximately 4 months (125 days) post-disease onset. A total of 95.5% and 88.1% were anti-N protein Ig positive after approximately 7 (203 days) and 10 months (304 days), respectively. After 18 months (548 days), 70.1% of the seroconverted individuals without reinfection remained positive for N-specific Ig.

**TABLE 2:** Anti-N protein Ig seropositivity over time in individuals without reinfection

Days from disease onset to blood sampling Median (IQR^*b*^)	Seropositivity (*n* = 67)^*a*^
27 (25–30)	94%
125 (110–132)	98.5%
203 (177–221)	95.5%
304 (267–313)	88.1%
548 (530–573)	70.1%

^*a*^ Two individuals without a positive N response at any time post-infection were excluded.

^*b*^ IQR, interquartile range.

## DISCUSSION

Since only S protein-based vaccines are approved and currently used in Europe and the USA, it is possible to classify individuals as previously infected using an anti-N antibody-based test. These measurements are relevant since antibody detection can complement RT-PCR for the diagnosis of COVID-19 and provide important knowledge in seroprevalence studies. Furthermore, it is of interest to enable the identification of previous infection, given that a natural infection could be assumed to boost vaccine responses (3). The use of an N-based immunoassay could help guide health authorities in prioritizing timing, and assessment of need, for booster vaccinations. At least since natural immunity appears to offer equal or greater protection against SARS-CoV-2 transmission compared to the immunity arising from vaccination alone (24). There is additionally increasing evidence for a relatively rapid waning of immunity against SARS-CoV-2 transmission by vaccination, whereas the waning after the infection seems more moderate (25). It is becoming clear that hybrid immunity, established by a combination of infection and vaccination, provides the utmost protection against subsequent COVID-19 infection compared to vaccination or infection alone (25 – 27). Regarding COVID-19 vaccination strategy, WHO does not recommend pre-vaccination screening for past infections (28), similar to other mass vaccination programs (such as the measles) where past infections do not exclude individuals from vaccination. It is considered beneficial to provide boosters prior to periods with expected increased incidence, including the winter season, to individuals in the high-risk groups and to those whose last immunological challenge is unknown. This is in fact relevant since the prevalence of infection is known to have been largely underestimated in many places throughout the pandemic (29). The durability of natural and hybrid immunity is unknown, as is the threshold of antibody protection. It has nevertheless been suggested that SARS-CoV-2 antibody levels correlate with protection (30, 31).

Anti-N protein antibody assays are valuable to detect a previous SARS-CoV-2 infection. It has nevertheless been debated whether antibodies against N protein constitute a more unreliable estimate of previous infection than S protein/RBD responses due to the more rapid waning of the N-specific Ig (32). Several studies investigating the N protein-specific humoral response have reported a rapid decline in anti-N protein seropositivity within a year post-infection (17, 18, 33). Different commercial anti-N assays were employed for these measurements, differing in being either qualitative or quantitative. Furthermore, most published anti-N protein antibody assays are based on the direct immunoglobulin detection principle (11, 12). Our newly developed ELISA is based on the sandwich principle where recombinant N protein is used both for capture and detection, similar to the Elecsys Anti-SARS-CoV-2 N protein assay from Roche Diagnostics, which could increase the assay sensitivity. The developed N-specific S-ELISA did reveal an increased sensitivity and specificity compared to the direct setups detecting N-specific IgG, IgM, and IgA. IgG is expected to be the major contributor upon measuring total N-specific Ig in serum, also in longitudinal samples in particular, but IgA appears to be present too. Very few individuals had detectable IgM levels during analysis of the 215 PCR-positive samples used for the validation of the direct ELISA setup.

To our knowledge, the newly developed S-ELISA assay is the first published open-platform antigen-sandwich anti-N protein assay. However, we have experienced that recombinant N protein is “sticky,” which may have hampered others in successfully establishing sandwich anti-N protein antibody assays. Thus, optimizing blocking conditions as described in the Materials and Methods section was important to minimize unspecific binding to make the S-ELISA applicable. It is important to note that the newly developed S-ELISA is a qualitative assay and not a quantitative assay. The assay was performed with high sensitivity and specificity and was comparable to the established Elecsys Anti-SARS-CoV-2 N protein assay, which in contrast to the new assay requires highly specialized and expensive equipment. A study using the Elecsys pan Ig platform in a prediction model indicated that natural infection might be detected beyond 500 days (34). This aligns with the results we observed in this study, where 70.1% of the seroconverted individuals with no reinfection remained seropositive for N-specific Ig for 550 days post-infection. Moreover, a recent study has in a similar manner evaluated N protein antibody titer longevity, also using the Elecsys pan Ig platform (35). They found that 80% were N positive 0–269 days post-infection, compared to the 88.1% (267–313 days post-infection) reported here. However, in contrast to our results, they observed that age and sex influenced antibody dynamics.

To study the antibody response kinetics, we constructed models using consecutive serological data from a population of SARS-CoV-2-infected individuals from the Faroe Islands (21). We modeled the Ig response against N protein and RBD and provided measurements of Ig levels up to 18 months after disease onset. The discrimination between vaccination- and infection-induced antibody responses became evident when we compared the waning of N versus RBD-specific Ig. The employed assays measure total Ig against N protein or RBD present in the samples using a single dilution, which offers simple, sensitive, and specific measurements suitable for screening of large cohorts. It should nevertheless be kept in mind that there is a small portion of individuals who do not develop antibodies against SARS-CoV-2 post-infection (36). This phenomenon is also observed following infection and vaccination for other infections, with influenza as a given example (37). Antibody non-responsiveness can be influenced by factors such as age, sex, comorbidity, prior immunity, and genetics, among others (37). In this study, a few individuals had no positive Ig response against N protein or/and RBD (at least until vaccination), which could be due to either false-positive testing or lack of seroconversion. Our data show that N-specific Ig exhibits steady waning after approximately 200 days in those individuals without reinfection, unaffected by vaccination, which aligns with what has been observed for anti-RBD Ig response previously (21, 22). The anti-RBD Ig response, as expected, was boosted by the vaccination. We defined the reinfection status solely by the presence or absence of a second positive RT‐PCR test. However, it should be noted that the data suggest that a few individuals within the non-reinfected group possibly could have had asymptomatic reinfection because of increased levels of anti-N protein Ig at the final blood collection. Since we observed an N protein Ig positivity drop during the 18 mo observation period to around 70%, we foresee continuous waning over time. This limits how long the N protein sandwich Ig assay can be used as a reliable marker post-infection. Similar tendencies have been shown for the total RBD Ig response in convalescent unvaccinated individuals (38). A limitation of the study includes the number of individuals within the vaccination group. Few individuals were unvaccinated (14%), and almost all unvaccinated individuals became reinfected, leading to some uncertainties when including this variable in the model due to lack of power. In addition, there is a gap in the sample collection between 350 and 500 days, which may introduce bias to the modeling curve. As a perspective, it should be mentioned that surely other antibody targets can be of interest in relation to SARS-CoV-2 surveillance and disease verification (39). This includes ORF8 antibodies as an example (40, 41).

In summary, we have developed a robust and sensitive assay for total anti-N protein Ig detection that is flexible, open, and affordable. A significant finding was that the N protein-specific total Ig response of the SARS‐CoV‐2 lasted, in the majority of the individuals, for 18 months post-infection. This shows the robustness of using anti-N protein Ig as a marker of previous SARS-CoV-2 infection over an extended period of time.

## Data Availability

The data that support the findings of this study are available from the corresponding author upon request.

## SUPPLEMENTAL MATERIAL

Supplemental file 1 spectrum.01796-23-s0001.tif Fig. S1.

Supplemental file 2 spectrum.01796-23-s0002.tif Fig. S2.

Supplemental file 3 spectrum.01796-23-s0003.tif Fig. S3.

Supplemental file 4 spectrum.01796-23-s0004.tif Fig. S4.

Supplemental file 5 spectrum.01796-23-s0005.docx Supplemental tables and legends.

ASM does not own the copyrights to Supplemental Material that may be linked to, or accessed through, an article. The authors have granted ASM a non-exclusive, world-wide license to publish the Supplemental Material files. Please contact the corresponding author directly for reuse.
