# Supplementary figures and images for: Distinguishing SARS-CoV-2 infection and vaccine responses up to 18 months post-infection using nucleocapsid protein and receptor-binding domain antibodies
Source: Microbiol Spectr. 2023 Sep 22;11(5):e01796-23. doi: 10.1128/spectrum.01796-23 (PMC10580960; doi:10.1128/spectrum.01796-23)

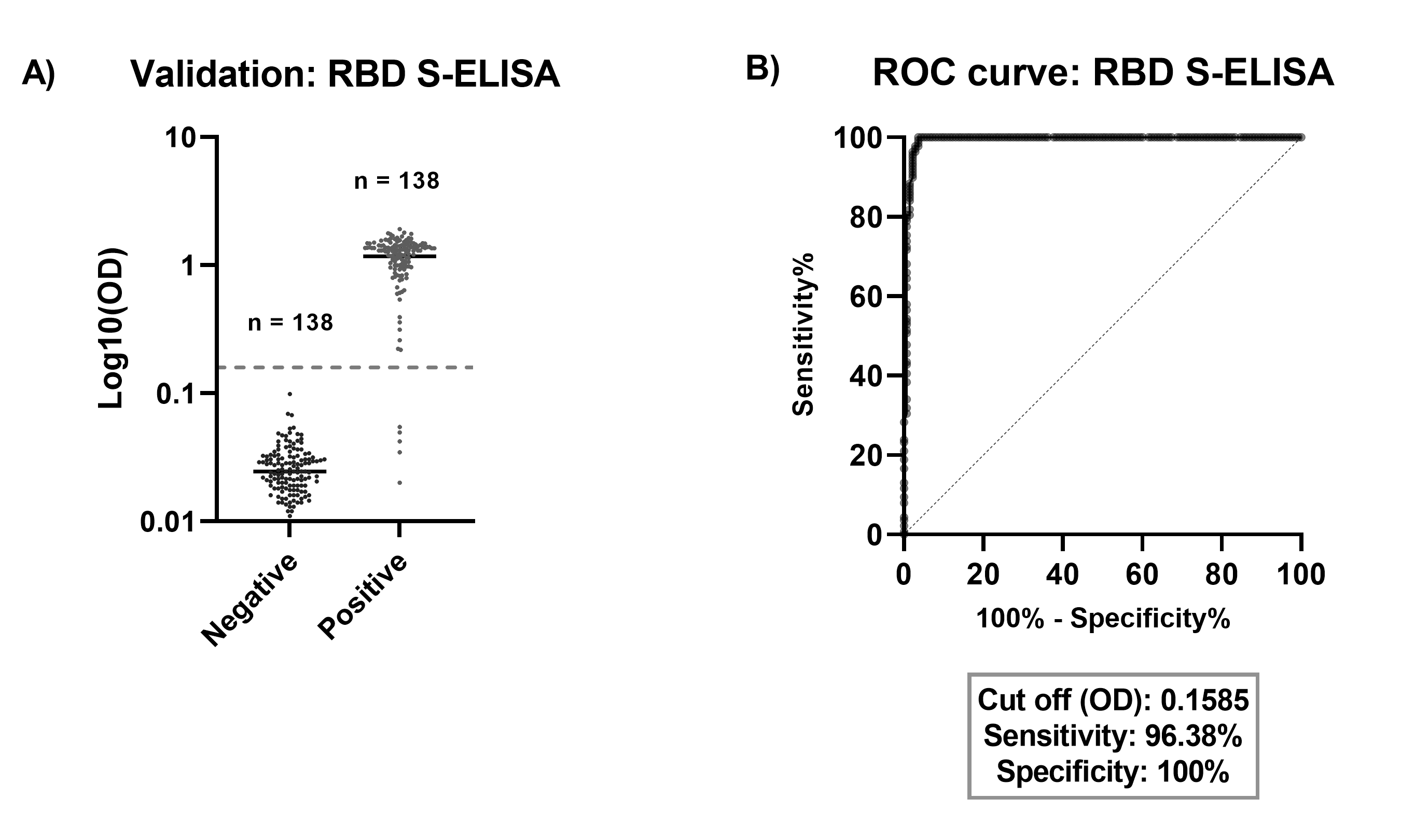

Supplement: Supplemental file 1 — Fig. S1. [file spectrum.01796-23-s0001.tif]

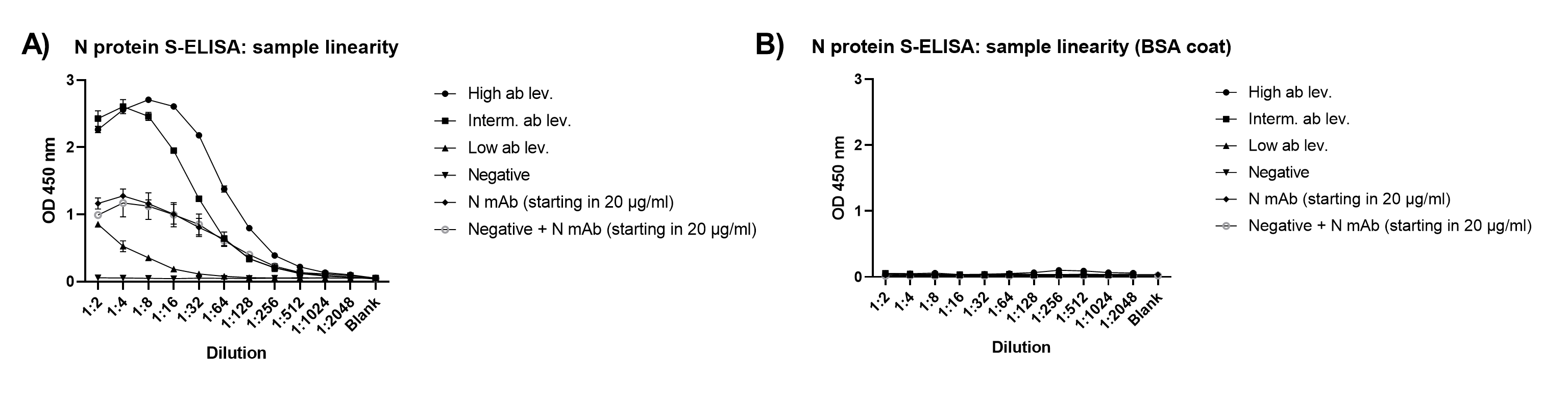

Supplement: Supplemental file 2 — Fig. S2. [file spectrum.01796-23-s0002.tif]

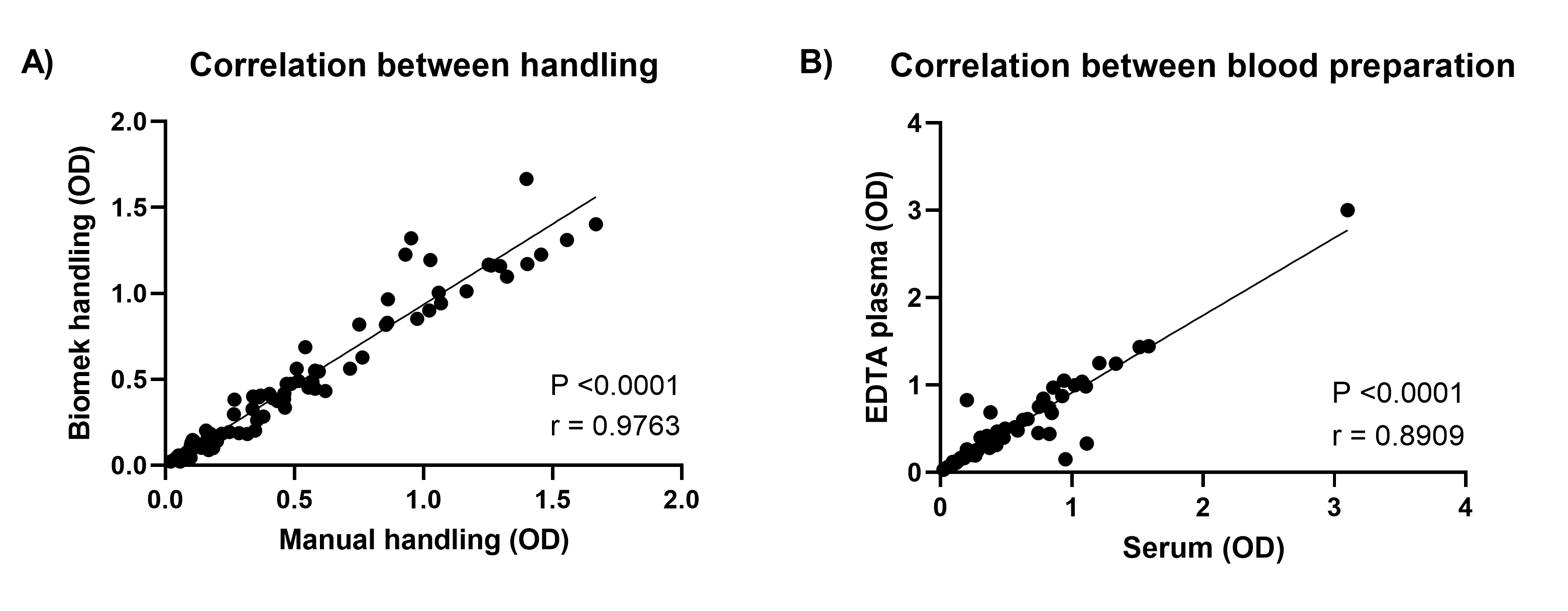

Supplement: Supplemental file 3 — Fig. S3. [file spectrum.01796-23-s0003.tif]

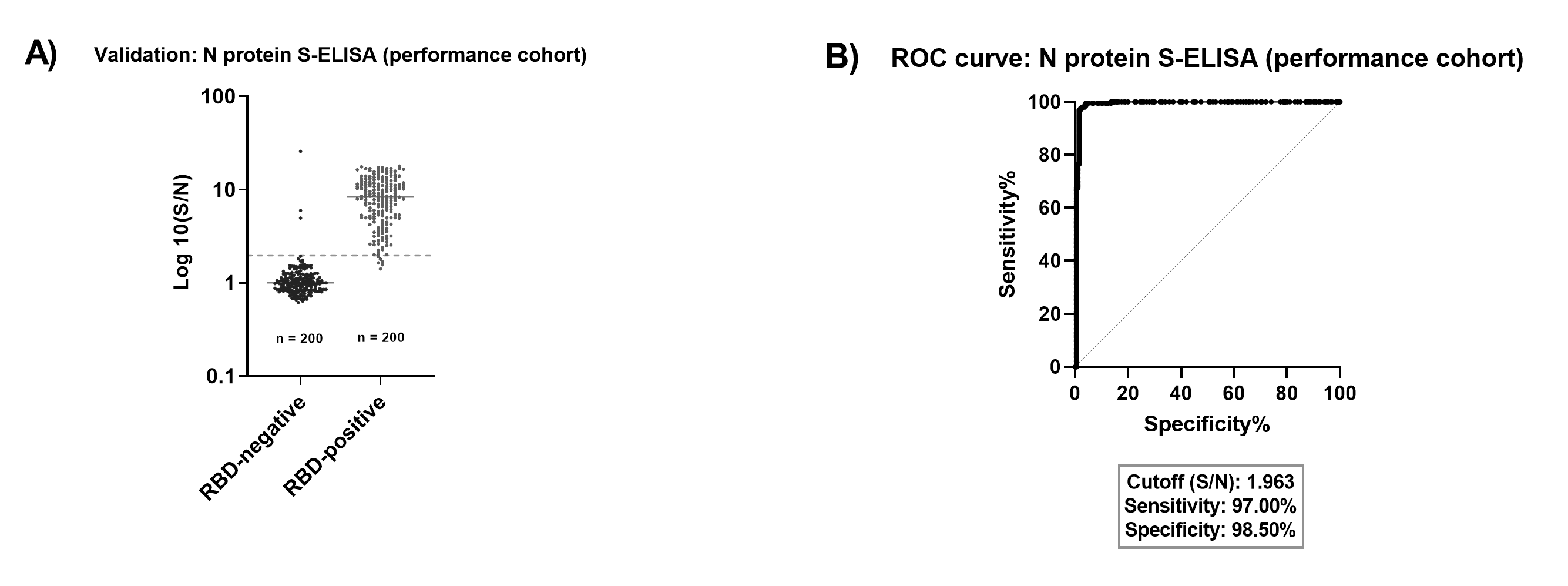

Supplement: Supplemental file 4 — Fig. S4. [file spectrum.01796-23-s0004.tif]
