# Supplementary material for: Distinguishing SARS-CoV-2 infection and vaccine responses up to 18 months post-infection using nucleocapsid protein and receptor-binding domain antibodies
Source: Microbiol Spectr. 2023 Sep 22;11(5):e01796-23. doi: 10.1128/spectrum.01796-23 (PMC10580960; doi:10.1128/spectrum.01796-23)
Supplement: Supplemental file 5 — Supplemental tables and legends. [file spectrum.01796-23-s0005.docx]

## Supplementary tables and legends

**Supplementary table 1: Overview of cohorts**

|  | **Validation cohort  (cohort I)** | **Performance cohort  (cohort II)** | **Study cohort  (cohort III)** |
| --- | --- | --- | --- |
| **Aim** | Assay validation and assessment of assay correlation between the new and a commercial assay | Assessment of assay correlation between the new and established RBD assay | Long-term evaluation of N- and RBD-specific immunity |
| **Sample origin** | Previously PCR confirmed SARS-CoV-2 infected healthcare professionals from Rigshospitalet and Herlev-Gentofte University Hospital and pre-pandemic negative controls | Medicals students from the University of Copenhagen | Previously SARS-CoV-2 infected individuals from The Faroe Island |
| **n** | 750  (375 positive and 375 negative) | 400  (200 anti-RBD positive and 200 anti-RBD negative) | 100 individuals with 5 consecutive samples |

**Supplementary table 2: Inter- and intra-assay variation in the newly developed anti-N S-ELISA**

|  | **CV%** |
| --- | --- |
| **Inter-assay variation  (n = 45)** | **2.02** |
| **Intra-assay variation** |  |
| Sample 1 | 8.24 |
| Sample 2 | 3.67 |
|  | **5.95** |

CV: Coefficient of variation

**Supplementary Figure 1: Assay validation of RBD S-ELISA**The RBD S-ELISA was validated using 138 PCR confirmed SARS-CoV-2 infected individuals and 138 SARS-CoV-2 RBD negative controls, which were subjected to antibody detection against spike RBD (A). ROC curve analysis was used to evaluate the performance of the RBD specific sandwich-ELISA setup detecting total Ig (B). Horizontal dashed line represents the positivity threshold.

**Supplementary Figure 2: Assay linearity**
The assay linearity was evaluated for three serum samples containing high, intermediate and low levels of N-specific antibodies, and for an in-house produced monoclonal mouse antibody against full-length N protein - both independently and spiked into a pre-pandemic negative control (A). The same experiment was performed using an unspecific coat of BSA (B). Error bars represent the SD of two independent experiments.

**Supplementary Figure 3: Parallelism between assay conditions**
The parallelism between the assay handling (A) and blood preparation (B) was evaluated using a Spearman Rank Correlation test and p <0.05 was considered significant.

**Supplementary Figure 4: Accordance between the N and RBD sandwich ELISA**
The accordance between the N protein S-ELISA and already established RBD S-ELISA was evaluated by using 200 SARS-CoV-2 RBD positive individuals and 200 SARS-CoV-2 RBD negative controls (A). ROC curve analysis was used to evaluate the performance of the newly established specific sandwich-ELISA using the performance cohort (B). Horizontal dashed line represents the positivity threshold. S/N: Signal-to-noise.
